# Boldine Treatment Induces Cytotoxicity in Human Colorectal Carcinoma and Osteosarcoma Cells

**DOI:** 10.7759/cureus.48126

**Published:** 2023-11-01

**Authors:** Panigrahi Chandan, Arora Dev, Devaraj Ezhilarasan, Karthik Shree Harini

**Affiliations:** 1 Dentistry, Saveetha Dental College, Saveetha Institute of Medical and Technical Sciences, Chennai, IND; 2 Pharmacology, Saveetha Dental College, Saveetha Institute of Medical and Technical Sciences, Chennai, IND

**Keywords:** apoptosis, cytotoxicity, boldine, osteosarcoma, colorectal cancer

## Abstract

Introduction

Cancer continues to be a significant health issue worldwide, with colorectal cancer (CRC) standing out as one of the most prevalent forms of cancer on a global scale. The lifetime risk of developing CRC is about one in 23 (4.3%) for men and one in 25 (4.0%) for women. Moreover, children and adolescents are frequently reported with osteosarcoma with a low five-year survival rate (69% and 67%, respectively).

Aim

The aim of the study was to analyze the cytotoxic effects of boldine against human CRC (HCT-116) and osteosarcoma cell lines (Saos-2).

Materials and methods

HCT-116 and Saos-2 cell lines were subjected to different concentrations of boldine treatment (5, 10, 20, 30, 40, and 50 μg/mL) and (10, 20, 40, 60, and 80 µg/mL), respectively, for 24 hours. The cytotoxicity was analyzed by MTT assay, AO/EB staining, DCFH-DA assay, and scratch assay.

Results

The MTT assay, microscopic analysis, and staining showed that boldine had dose-dependent cytotoxic effects against HCT-116 and Saos-2 cell lines by inhibiting their proliferation, viability, and migration, and inducing ROS-mediated apoptosis.

Conclusion

The study concluded that boldine had a concentration-dependent cytotoxic effect on human CRC and osteosarcoma cell lines.

## Introduction

Boldine is a natural alkaloid normally found in the leaves of Peumus boldus molina [1]. It is classified under the family of aporphine alkaloids [2]. Several plants and plant derivatives, including flavonoids and alkaloids, have been identified for their biological properties such as anti-inflammatory, antioxidative, analgesic, and antiallergenic activities [3]. Boldine therapy has been recognized as an herbal therapy in various parts of the world, mainly in South America and Europe. Boldine infusions are applied traditionally in folk medicine and used as a medicinal plant for the treatment of hepatobiliary disorders and exhibiting immunomodulating, smooth muscle relaxing, and neuroleptic-like properties [4]. Boldine is known for its ability to inhibit oxidative damage in the liver, hepatic microsomes, and erythrocytes by preventing free radical-induced lysis of intact hepatocytes and red blood cells [5]. Additionally, boldine is recognized for its cytoprotective and anticancer properties.

According to the World Health Organization (WHO), cancer was the second leading cause of death worldwide in 2018. In 2020, about 9,503,710 (49.3%) new cancer cases were estimated all over Asia, 4,398,443 (22.8%) in Europe, and 2,556,862 (13.3%) in North America. Between ten and 20 lakh new cases of colorectal cancer (CRC) are diagnosed each year, making it one of the most prevalent cancers in the world. With over 700,000 cancer-related deaths each year, only lung, liver, and stomach cancers contribute to most of these cases [6]. CRC is the second most prevalent cancer among women (9.2%) and males (10%), respectively. Most CRC cases are found in Western nations (55%); however, this tendency is shifting as a result of other nations' rapid recent progress [7]. Particularly, the incidence rate of DRC in India has increased by 20% from 2004 to 2014. Moreover, India has a very low survival rate of 40%. Genetic mutations are one of the major risk factors of CRC. Only 5% of CRC cases are caused by inherited malignancies. Age is another key risk factor for CRC: after the fifth decade of life, the risk of getting CRC is significantly raised, when compared with those under the age of fifty (apart from inherited cancers) [8]. CRC is more likely to occur in people with a personal history of CRC or inflammatory bowel disease (IBD). Changes in one’s eating and exercise routines can lower other lifestyle-associated risk factors. Diagnosis of CRC usually involves colonoscopy; however, novel biomarkers such as DNA, RNA, and proteins are recently identified. Treatment for most CRC patients with metastatic disease typically consists of cytotoxic and targeted biological therapies. Fluoropyrimidines (such as 5-fluorouracil or capecitabine) are used as the first-line chemotherapy for palliative purposes, either alone or in combination with leucovorin and other cytotoxic drugs including oxaliplatin and capecitabine or irinotecan. Leucovorin is used to lessen the side effects of treatment; conversely, other cytotoxic drugs have been demonstrated to improve response rates and progression-free survival while simultaneously causing the side effects of the treatment to be more severe [9].

Osteosarcoma, a malignant bone condition, is a type of bone cancer, commonly found in long bones. It mostly occurs in teenagers and adults. The incidence rate among children under 15 years of age is 5.6 per million. In India, the incidence ranges from 4.7% to 11.6 %. Several factors including ionizing radiation exposure, genetic conditions, existing bone disease, and hereditary retinoblastoma increase the risk of osteosarcoma. Diagnosis of the disease requires radiography and MRI (magnetic resonance image) of the bone. Treatment mostly involves chemotherapy (methotrexate and cisplatin), surgery, and radiation therapy [10]. Symptoms of osteosarcoma mainly include swelling, joint pain, and bone injury. There are three grades of osteosarcoma: low, intermediate, and high [11]. Although several therapeutic strategies are available, noninvasive treatment with an effective drug having no or fewer side effects would be a significant development in the treatment of osteosarcoma. Saos-2 is a commonly used osteosarcoma cell line for performing in vitro studies [12]. Therefore, the objectives of the current study were to investigate the cytotoxic potential of boldine, an herbal compound against human CRC and osteosarcoma, to analyze the ability of boldine to prevent cancer cell survival, proliferation, and migration and to elucidate the apoptotic mechanism of boldine-induced cell death in CRC and osteosarcoma cell lines.

## Materials and methods

Reagents

Boldine was purchased from Sigma-Aldrich, Burlington, MA. The necessary chemicals for cell culture including Dulbecco’s minimum-low glucose medium (DMEM), penicillin, streptomycin, trypsin-EDTA, fetal bovine serum, and 3-(4,5-dimethylthiazol-2-yl)-2,5-diphenyltetrazolium bromide (MTT) were purchased from GIBCO BRL. Other chemicals of analytical grade used in various assays were purchased locally.

Cell culture 

Human CRC (HCT-116) cell lines and human osteosarcoma (Saos-2) cell lines were procured from the National Centre for Cell Science (NCCS), Pune, India, and cultured at the standard conditions of 5% CO2 and 37 °C. DMEM with 10% FBS, penicillin (100 units/mL), and streptomycin (100 μg/mL) were used to culture the cells. HCT-116 cells were treated with 5, 10, 20, 30, 40, and 50 μg/mL of boldine for 24 hours. Saos-2 cells were treated with 10, 20, 40, 60, and 80 μg/mL of boldine for 24 hours. HCT-116 and Saos-2 cells without boldine treatment served as control.

MTT Assay

The cultured HCT-116 and Saos-2 cells were treated with different concentrations of boldine for 24 hours, and the media was replaced with an MTT reagent. After four hours of incubation at 37 °C, the purple-blue-colored formazan crystals were dissolved in dimethyl sulfoxide (DMSO), and the absorbance was measured at 570 nm [13]. Morphological changes were observed under a microscope [14]. With the results of the MTT assay, the dose of boldine for the subsequent experiments was fixed depending on the inhibitory concentrations. 

Acridine Orange/Ethidium Bromide Staining

HCT-116 and Saos-2 cells were treated with 30 μg/mL and 60 μg/mL boldine, respectively, for 24 hours, and centrifuged and harvested. The cells were then fixed in 70% ethanol. Staining was performed using acridine orange and ethidium bromide (AO/EB). The stained cells were examined using a fluorescent microscope [15]. The staining was performed in triplicates. 

Dichlorodihydrofluorescein Diacetate Staining

After 24 hours of boldine (30 μg/mL) treatment, the HCT-116 cells along with control cells were treated with DCFH-DA (2, 7-dichlorodihydrofluorescein diacetate) and incubated for 30 minutes at 37 °C. Fluorescence was measured at 485 ± 10 nm and 530 ± 10 nm [16]. The staining was performed in triplicates.

Scratch Assay

Saos-2 cells were cultured in six-well plates to confluence. A sterile microtip was used to make a scratch in the monolayer of cells. The cells were cultured for 24 hours with 60 μg/mL of boldine. After the treatment, cells were observed using a phase-contrast microscope.

Statistical analysis

The results were expressed as mean ± SD. Statistical significance was evaluated by Dunnett’s test and a one-way ANOVA. P value < 0.05 was considered significant.

## Results

**Figure 1:**
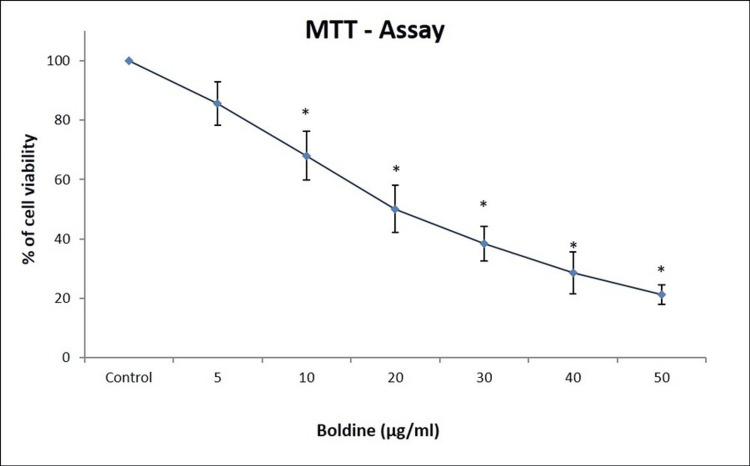
Boldine treatment suppresses the proliferation of HCT-116 cells A dose-dependent decrease in the proliferation and viability of HCT-116 cells upon boldine treatment in MTT assay. Data are shown as means ± SD (n = 3) compared with the control group, p < 0.001. HCT-116 cells, human colorectal carcinoma cell line; MTT, 3-(4,5-dimethylthiazol-2-yl)-2,5-diphenyltetrazolium bromide.

**Figure 2:**
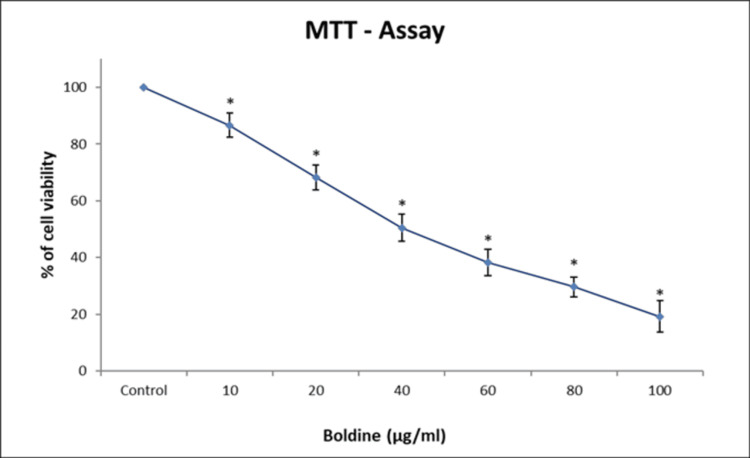
Boldine treatment suppresses the proliferation of Saos-2 cells A dose-dependent decrease in the proliferation and viability of Saos-2 cells upon boldine treatment in MTT assay. Data are shown as means ± SD (n = 3) compared with the control group, p < 0.001. Saos-2 cells, human osteosarcoma cell line; MTT, 3-(4,5-dimethylthiazol-2-yl)-2,5-diphenyltetrazolium bromide.

To evaluate the effect of boldine on the viability and proliferation of HCT-116 and Saos-2 cells, an MTT assay was performed. The cytotoxic effects of boldine were analyzed by treating the HCT-116 cells with 5, 10, 20, 30, 40, and 50 μg/ml of boldine and Saos-2 cells with 10, 20, 40, 60, 80, and 100 μg/mL of boldine for 24 hours. Boldine treatment resulted in a significant decrease in the proliferation of HCT-116 and Saos-2 cells. A reduction in viability and proliferation of HCT-116 and Saos-2 cells was observed in a dose-dependent manner (Figures 1-2).

**Figure 3:**
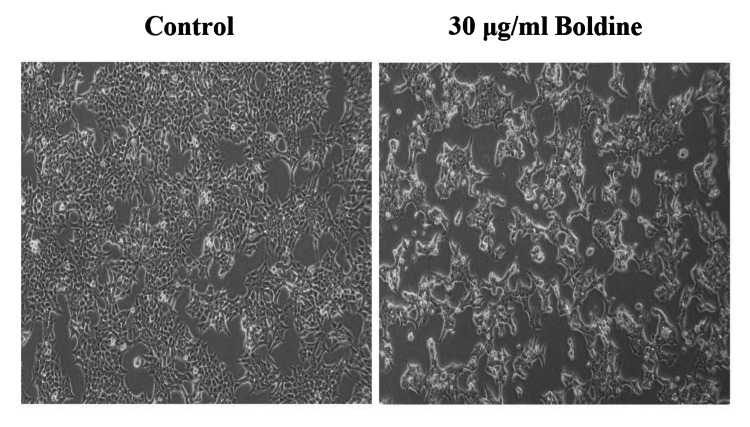
Boldine alters the morphology of HCT-116 cells Boldine treatment reduced the number of CRC cells and resulted in altered morphology under a phase contrast microscope (20x magnification). HCT-116 cells, human colorectal carcinoma cell line.

**Figure 4:**
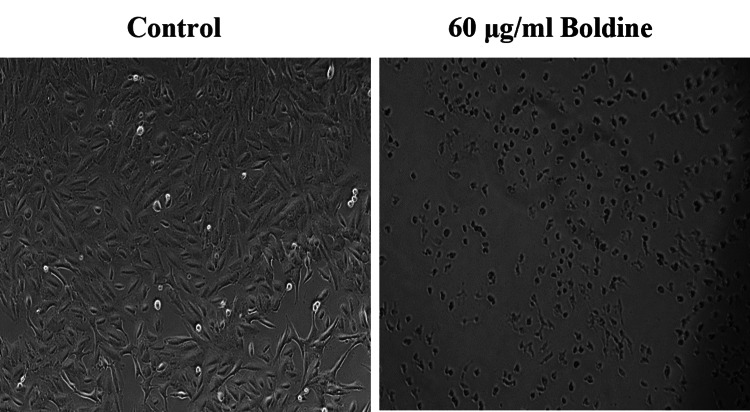
Boldine alters the morphology of Saos-2 cells Boldine treatment reduced the number of osteosarcoma cells and resulted in altered morphology under a phase contrast microscope (20x magnification). Saos-2 cells, human osteosarcoma cell line.

Microscopic evaluation of cytotoxicity revealed altered morphology and reduced number of HCT-116 and Saos-2 cells upon 30 μg/ml and 60 μg/mL boldine treatment, respectively (Figures 3-4).

**Figure 5:**
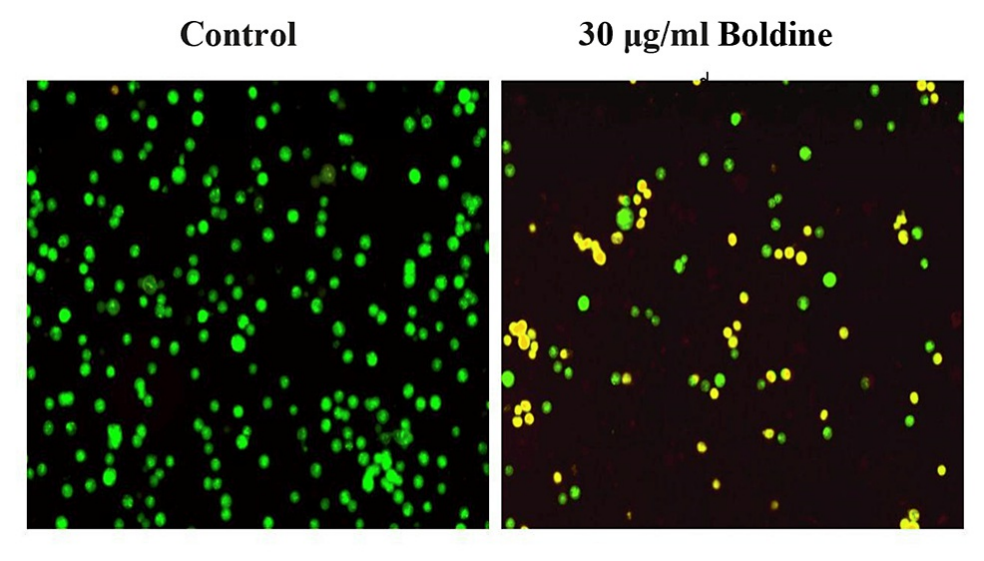
Apoptotic effect of boldine in HCT-116 cells Boldine increases the number of early and late apoptotic cells and necrotic cells in CRC cells, visualized by AO/EB staining. HCT-116 cells, human colorectal carcinoma cell line; AO/EB, acridine orange/ethidium bromide.

**Figure 6:**
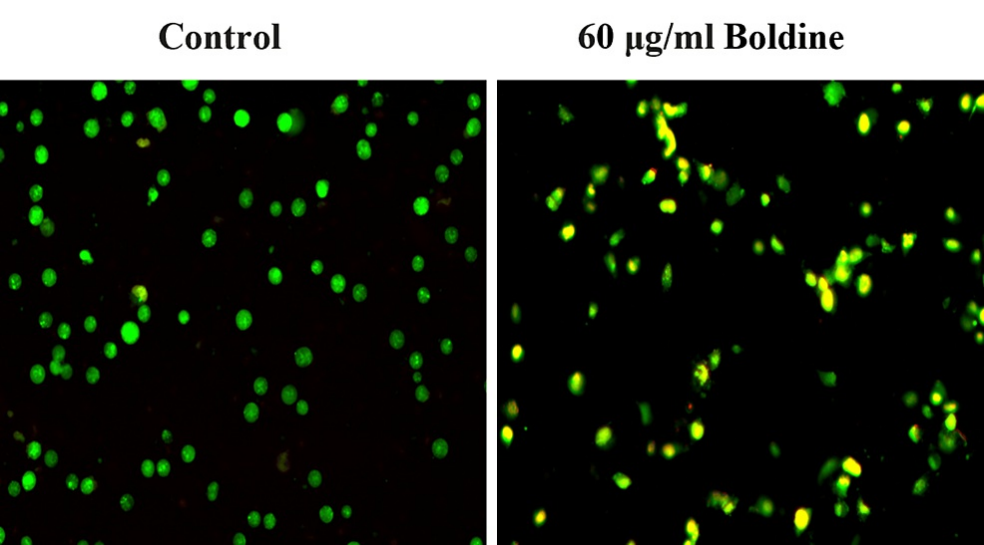
Apoptotic effect of boldine in Saos-2 cells Boldine increases the number of early and late apoptotic cells and necrotic cells in an osteosarcoma cell line, visualized by AO/EB staining. Saos-2 cells, human osteosarcoma cell line; AO/EB, acridine orange/ethidium bromide.

To determine whether the cytotoxicity is because of apoptosis of the cells, AO/EB staining was performed. The normal viable cells, early apoptotic cells, and late apoptotic cells were stained green, yellow, and orange, respectively. Boldine treatment significantly reduced the number of viable cells and increased the number of apoptotic cells. Therefore, boldine induced apoptosis in HCT-116 and Saos-2 cells (Figures 5-6).

**Figure 7:**
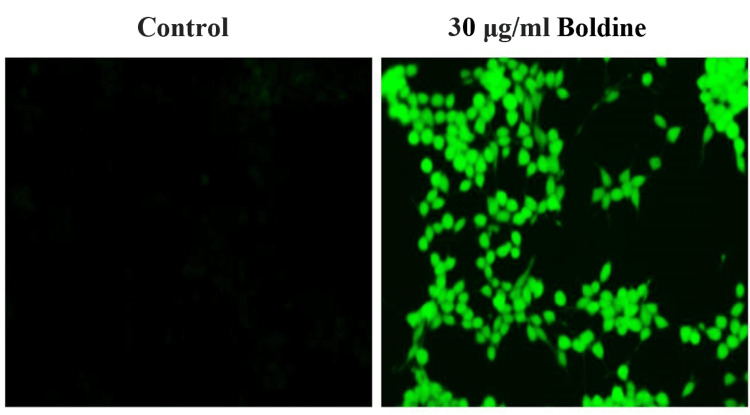
Effect of boldine on ROS generation in HCT-116 cells Boldine significantly increased the production of intracellular ROS in CRC cells, visualized under fluorescent microscopy (20x). HCT-116 cells, human colorectal carcinoma cell line; ROS, reactive oxygen species.

To elucidate the mechanism-causing apoptosis of HCT-116 cells, the intracellular reactive oxygen species (ROS) generation was analyzed. The ROS production was evident from the green fluorescence emitted. Boldine treatment significantly increased the ROS levels in HCT-116 cells, increasing the intracellular oxidative stress, and ultimately resulting in apoptosis (Figure 7).

**Figure 8:**
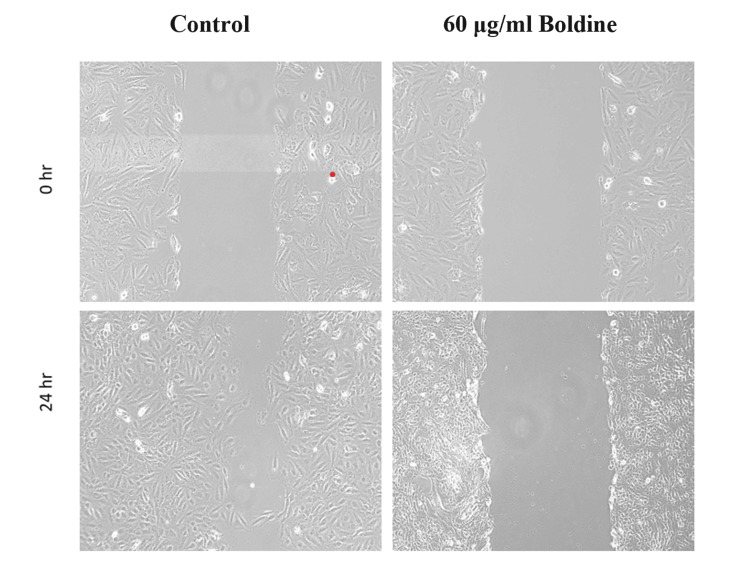
Effect of boldine in the migration of Saos-2 cells The image represents scratch assay, and boldine inhibits the migratory ability of osteosarcoma cells (20x magnification). Saos-2 cells, human osteosarcoma cell line.

Scratch assay was also done to analyze cell migration. The results of the scratch assay observed by phase contrast microscopy revealed that boldine suppressed the migratory activity and wound healing ability of Saos-2 cells over 24 hours (Figure 8).

According to these results, it can be concluded that boldine can be a potent anticancer drug against human CRC (HCT-116 cells) and osteosarcoma (Saos-2 cells).

## Discussion

While early detection and treatment of cancer can enhance the survival rates of patients, individuals in developing countries and rural areas often resort to traditional medicines because of limited access to modern diagnostic tools and treatments [17]. Almost 80% of people worldwide are reported to use traditional medications, including phytomedicine, according to the World Health Organization. In particular, herbs and herbal compounds are being tested and used for their various pharmaceutical properties against cancer with lesser side effects. Nature has always been an invaluable source of products that can serve as therapeutic agents [18]. In the continuing efforts to identify new candidates for cancer chemotherapeutic/preventive effects among plant secondary metabolites, boldine, an aporphine from the Chilean boldo tree (P. boldus), exhibited potent inhibition of cancer cell growth [19].

Earlier studies have reported the anticancer effect of boldine, in vitro and in vivo. Boldine administration was shown to slow down the tumor growth and increase the survival of mice with mammary carcinoma, post-treatment [20]. Boldine inhibited the viability and proliferation of the human breast cancer cell line, MCF-7, by the activation of the mitogen-activated protein kinase pathway [21]. The induction of apoptosis and cell cycle arrest by boldine exhibited a potential therapeutic effect against bladder cancer [22]. Therefore, boldine was tested for its cytotoxicity against HCT-116 and Saos-2 cells in this current study. Further, the mechanism underlying the cytotoxicity of boldine was also investigated.

Apoptosis is a mode of programmed cell death that occurs to eliminate malignant or abnormal cells and maintain the cell population through a variety of molecular mechanisms. However, cancer cells evade apoptosis, proliferate rapidly, and exhibit resistance to anticancer treatments [23]. Primarily, an MTT assay was performed to analyze the ability of boldine to inhibit the proliferation of HCT-116 and Saos-2 cells. The assay is based on the enzymatic reduction of MTT to formazan. MTT assay is a quantitative analysis that measures the cell growth rate, which is related to the absorbance [24]. On that account, the study results show that boldine significantly represses the proliferation and viability of HCT-116 and Saos-2 cells. Further, the cytotoxicity of boldine was confirmed by examining cells under a phase-contrast microscope. Boldine reduced the number of HCT-116 and Saos-2 cells and altered the morphological structure of cancer cells.

To determine whether the cytotoxicity of boldine was because of apoptosis, AO/EB staining was performed. Apoptosis-associated changes in the cell membrane can be detected under a fluorescent microscope by AO/EB staining [25]. This staining technique is used to differentiate between normal cells, early and late apoptotic cells, and necrotic cells. AO/EB staining is a standard method for detecting apoptosis, both qualitatively and quantitatively [26,27]. The principle is based on the ability of AO to penetrate normal cells with intact membranes, resulting in green fluorescence. Conversely, EB penetrates apoptotic cells with compromised membranes, resulting in orange-red fluorescence [28]. The results of this study revealed increased apoptotic and necrotic cells upon boldine treatment.

The underlying mechanism of apoptosis of cancer cells was analyzed. It is established that an imbalance in the redox state is toxic to the cells that cause cell death. ROS-mediated apoptosis of HCT-116 cells in this study suggested that the apoptosis of CRC cells was mediated by increased intracellular oxidative stress. 

The scratch assay is an in vitro technique used to evaluate cell migration. The assay is used to evaluate therapeutic compounds before they are used in clinical trials because metastasis is one of the important properties of cancer cells. Cell migration is essential for the development and maintenance of multicellular organisms, but abnormal cell migration is found in many pathological disorders such as cancer [29]. Previous studies show that herbal compounds significantly inhibit the migration of cancer cells. On that account, in an in vitro study, boldine at increasing concentration inhibited the metastasis of the HSC-4 oral squamous cell carcinoma cell line in a time-dependent manner [30]. Similarly, the present study also showed that boldine suppressed the migratory and wound-healing ability of Saos-2 cells.

Limitations

As cancer is a disease with extreme complexity involving several molecular signaling pathways, genetic instability, environmental impact, and so on, a therapeutic drug with a clear understanding of the mechanism of action and its targets would aid in efficient anticancer treatment. Although this present study emphasizes the antiproliferative, antimigratory, and apoptotic properties of boldine against CRC and osteosarcoma, the exact target and mechanism of action of boldine must be elucidated. Further, the ability of boldine to selectively act against multiple targets can be extensively studied for an effective cancer treatment.

## Conclusions

Boldine exhibited a concentration-dependent cytotoxic effect against HCT-116 and Saos-2 osteosarcoma cells by inhibiting cell viability and proliferation and promoting apoptosis. The study reveals the anticancer potential of boldine by virtue of its antiproliferative, oxidative stress mediated apoptotic, and antimigratory properties. Therefore, boldine can be further studied to be used as a potential drug candidate against human colorectal carcinoma and osteosarcoma.
